# New perspective on African swine fever: a bibliometrics study and visualization analysis

**DOI:** 10.3389/fvets.2023.1085473

**Published:** 2023-05-17

**Authors:** Zhengyu Yu, Li Xie, Peiqiang Shuai, Jing Zhang, Wei An, Miao Yang, Jing Zheng, Hua Lin

**Affiliations:** ^1^Department of Hematology, West China Hospital, Sichuan University, Chengdu, China; ^2^State Key Laboratory of Wildlife Quarantine and Surveillance (Sichuan), Technology Center of Chengdu Customs, Chengdu, China

**Keywords:** African swine fever, bibliometrics, citation, Web of Science, visualized analysis

## Abstract

**Introduction:**

African swine fever (ASF) is a contagious viral disease that can have devastating effects on domestic pigs and wild boars. Over the past decade, there has been a new wave of this ancient disease spreading around the world, prompting many scholars to dedicate themselves to researching this disease. This research aims to use bibliometric methods to organize, analyze and summarize the scientific publications on ASF that have been amassed in the past two decades.

**Methods:**

This paper used VOSviewer, CiteSpace, and a bibliometric online analysis platform to conduct performance analysis and visualization studies on 1,885 academic papers about ASF in the Web of Science from January 2003 to December 2022.

**Results:**

The amount of literature published on ASF has increased exponentially in recent years, and the development trend of related research is good. A group of representative scholars have appeared in this research field, and some cooperative networks have been formed. *Transboundary* and *Emerging Diseases* is the journal with the most publications in this field, while *Virus Research* is the journal with the most citation per article. High-productivity countries are led by China in terms of the number of articles published followed by the United States and Spain. In regard to the average number of citations, the scholars in the UK are in the lead. The institution with the most articles was the Chinese Academy of Agricultural Sciences. The analysis of high-frequency keywords showed that the pathogens and epidemiology of ASF were the research hotspots in this field, and the research content was closely related to molecular biology and immunology. The burst keywords “transmission”, “identification”, “virulence”, “replication”, and “gene” reflects the research frontier. In addition, by collating and analyzing highly cited journals and highly co-cited references, we explored the knowledge structure and theoretical basis of this field.

**Discussion:**

This is the first bibliometric analysis report on ASF research, which highlights the key characteristics of ASF research and presents the research status and evolution trend in this field from a new perspective. It provides a valuable reference for further research.

## 1. Introduction

African swine fever (ASF) is an acute hemorrhagic fever caused by the African swine fever virus (ASFV), which is a large double-stranded DNA virus in the family *Asfarvirida* (1, 2). Hyperacute and acute forms of ASF result in nearly 100% mortality in domestic pigs (3, 4), and no reliable commercial vaccine is currently available (5–8). ASFV is hosted by both suids and soft ticks from the genus *Ornithodoro*s. It can be maintained for extended periods in *Ornithodoros* ticks and replicated to high titers (9). The existence of the virus in nature can be long-term and stable by the infection of warthogs and possibly other wild suids, which is difficult to eradicate from Africa (10).

The disease was first identified in East Africa in the early twentieth century (11), and it spread from Africa to Europe twice in 1957 and 1960 and then to the Americas. Outbreaks were not eradicated until the mid-1990s, except in Africa and Sardinia (12, 13). In 2007, ASF was first introduced to Georgia from sub-Saharan Africa (14) and then spread to the Russian Federation and the European Union (15–17). For the first time, ASF also spread to China, a major pork producer and consumer, in 2018 (18, 19). In addition, cases of infection have also been detected in Southeast Asia and other parts of Asia (20). In 2021, ASF reappeared in the Americas (21, 22). Although the disease does not pose a threat to human health, due to its high mortality rate to domestic pigs, large-scale culling operations covered by disease control regulations, restrictions on pork imports and exports, and many other factors, ASF has a serious impact on the global pig industry, people's livelihood, world trade, and food security. According to the forecast, if epidemics were to start in Denmark, there would be significant economic damage, with €12 million in direct costs and €349 million in export losses (23). If a piggery unit experiences an outbreak of ASF, it can expect to lose an estimated $910,836.70 per year (24). Therefore, ASF has been of high concern to scholars all over the world in recent years, and relevant research has also achieved fruitful results.

In this context, we used bibliometric methods to conduct a comprehensive collation of ASF research over the past two decades, analyzed the knowledge structure and quantitative information in the field, and provided performance analysis and visualization maps. The map was used to explore the internal relations between the information, and it showed the research focus and the evolution trend in recent years to provide a scientific reference for related research on ASF.

## 2. Methodology

### 2.1. Research methods

Bibliometrics is a quantitative research method that uses mathematical and statistical methods to review and describe published literature in a particular field (25). This research method can obtain and analyze important information such as the details of publication authors, keywords, journals, institutions, countries, and references. The results will help to understand the development trend of a scientific field, research focus, and researcher cooperation relationships (26). Furthermore, the use of computer technology to present results graphically and visually can help to uncover hidden relationships within the data and make the results more comprehensive (27).

Data visualization technology is a very important research method and means in bibliometrics. VOSviewer and CiteSpace are two commonly used data visualization analysis software programs. Using VOSviewer, a large-scale bibliometric map can be constructed to reflect the importance of items such as authors, keywords, institutions, and the strength of relationships with adjacent items through label views, density views, cluster density views and scatter views (28). CiteSpace is a Java application for analyzing and visualizing co-citation networks. It can divide a time interval into several time slices, from which individual co-citation networks can be obtained to highlight the main changes between adjacent time slices (29). Moreover, CiteSpace can detect and visualize trends and changes in science over time. It can be used to explore the dynamics of a profession, that is, the mapping of temporal changes from its knowledge base to the research frontier (30). Therefore, VOSviewer version 1.6.18 and CiteSpace version 6.1.R6 were used in this study to conduct a bibliometric analysis of ASF-related academic publications. In addition, BiblioShiny, a software package running in R language, and an online analysis platform of bibliometrics were used as complementary methods in this study.

### 2.2. Data sources

This study delimits the scope of analysis, employs a strict literature search strategy, and selects the Web of Science (WoS) as the data source. The WoS includes relevant material from a wide range of research fields and is a high-quality digital database that is widely accepted by researchers around the world (31). It exceeds other databases in functionality and complexity, with historically greater coverage (32).

**Figure 1:**
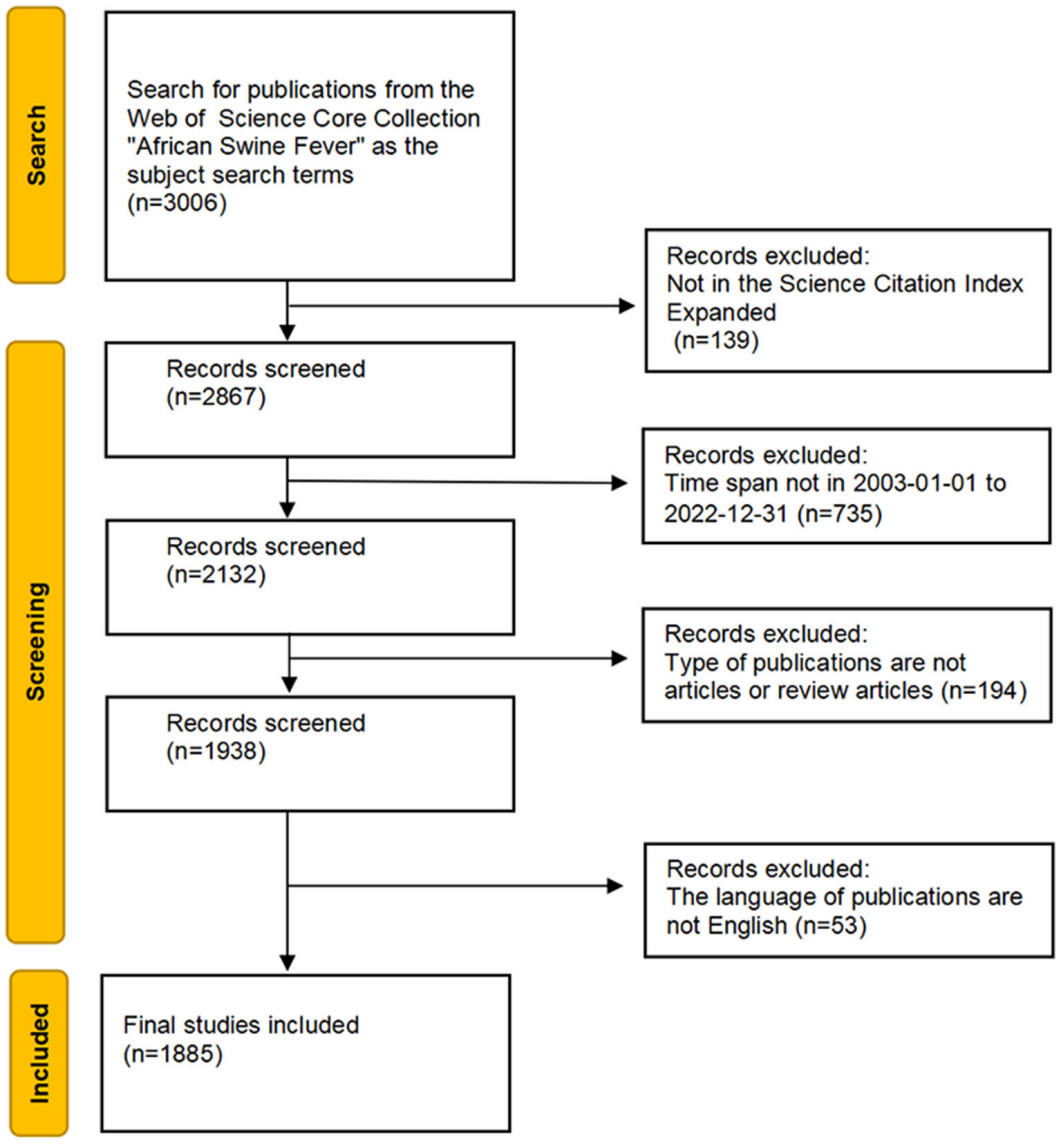
The publications selection process for this bibliometric analysis study.

The Web of Science Core Collection (WoSCC) was selected as the data source for this particular study. The Science Citation Index Expanded was chosen, and the retrieval strategy was to use “African swine fever” as the subject search term to guarantee the comprehensive and precise retrieval of data. Additionally, the time horizon was set from January 1, 2003, to December 31, 2022. The initial search identified 2,132 relevant articles, including all document types. Considering the completeness of the literature information, the literature types were selected as articles and review articles, and the language was restricted to English for ease of analysis. Finally, 1,885 valid papers were obtained. Retrieved publications were exported as plain text files with “full record and cited references”, which were uploaded to the VOSviewer and CiteSpace for analysis under the name “download_X.txt”. The selection process is detailed in Figure 1.

## 3. Results

### 3.1. Performance analysis

**Figure 2:**
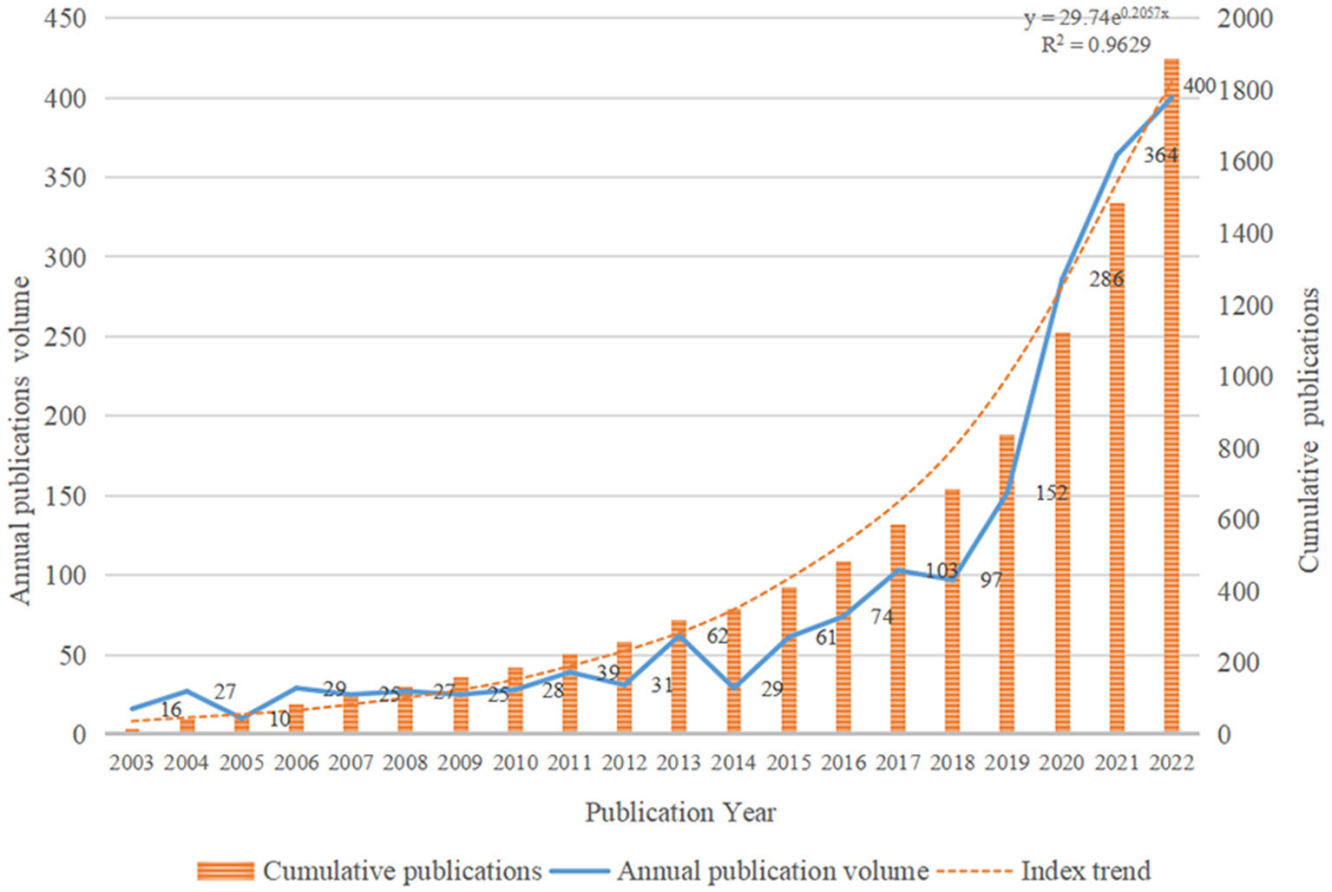
Distribution of publications on African swine fever (ASF) from 2003 to 2022.

The 1,885 articles used in this study come from 7,621 authors affiliated with 2,021 institutions in 111 countries/regions. These articles were published in 338 journals and cited 46,521 references from 12,358 journals. The number and the cumulative number of papers published in different periods can reflect the development trend of this research field. In the past two decades, the temporal distribution of 1,885 articles in the ASF research field is shown in Figure 2. Although the annual publication volume did not change much before 2013, which was < 40, on the whole, the number of publications in this field is on the rise. The number of publications has increased significantly, especially after 2018. More than 150 articles were annually published for four consecutive years from 2019 to 2022, and the increase was large. In 2018, ASF spread to China, the world's largest pig breeder, sparking a research boom among researchers in this field. Linear regression analysis was carried out on the publication time of the literature and the annual cumulative number of publications, *R*^2^ = 0.9629. The model fitting effect is good, which is in line with the scientific exponential growth law proposed by Price, that is, various scientific indicators increase approximately exponentially with time (33). This shows that the relevant research on ASF is currently in a period of rapid development, and the speed of achievement output and literature publication is also accelerating.

### 3.2. Analysis of prolific authors

To understand the representative scholars and main research teams, this paper studies the top 11 scholars with the number of publications, as shown in Table 1. Sandra Blome, a virologist from the Friedrich Loeffler Institute in Germany, published the most papers over the past two decades (75 articles, 3.98%, 25.37 average citation per article). Linda Dixon of the Pirbright Institute in the UK was second in the number of publications (64 articles, 3.40%, 64.66 average citation per article). Carmina Gallardo, a Spanish scholar from the European Union Reference Laboratory for ASF, ranked third (55 articles, 2.92%, 50.53 average citation per article). Based on the WoS, the H-index is a useful indicator to assess the impact and productivity of certain countries/regions, institutions, or scientists in an academic field (34). It can be simply expressed as H publications were cited no less than H times. For example, a scientist with an H-index of 30 means that he has 30 papers that have been cited at least 30 times. It has mostly become an objective measure of scientific achievement. According to statistics, the top 3 authors for the H-index in this study are Linda Dixon (H-index = 40), Carmina Gallardo (H-index = 34), and José Manuel Sánchez-Vizcaíno (H-index = 30). Dr. Sandra Blome is responsible for the Germany National Reference Laboratories for African and classical swine fever. Her research focuses on the pathogenesis of viral infectious diseases such as classical swine fever and ASF (35, 36), especially the mechanism of virus–host interaction and the development of vaccines (37). Dr. Linda Dixon's research focuses on the functional genomics of ASFV(1) and the mechanisms of immune evasion and pathogenesis (38), and her research team has also been committed to the research and development of effective vaccines (39). Dr. Carmina Gallardo's main expertise is in ASF research, mainly involving new diagnostic tools, molecular epidemiology, epidemic control strategies and vaccine research (40, 41). Professor Sánchez-Vizcaíno's research has been vital in the fight against various animal diseases, such as ASF, African horse sickness, and classical swine fever. His work has enabled the development of new techniques, quick and accurate diagnostic tools, innovative epidemiologic approaches, and novel vaccine strategies (42, 43).

**Table 1:** The top 11 authors with most publications related to African swine fever (ASF).

**Runk**	**Author**	**Publications**	**Percentage**	**Citations**	**Average citation**	**H-index**	**Location**
1	Blome, sandra	75	3.98	1,903	25.37	24	Germany
2	Dixon, linda k.	64	3.40	4,138	64.66	40	United Kingdom
3	Gallardo, carmina	55	2.92	2,779	50.53	34	Spain
4	Borca, manuel v.	44	2.33	1,316	29.91	20	United States
5	Gladue, douglas p.	43	2.28	1,270	29.53	19	United States
6	Sanchez-vizcaino, jose manuel	42	2.23	1,717	40.88	30	Spain
7	Beer, martin	34	1.80	1,186	34.88	17	Germany
8	Stahl, karl	34	1.80	939	27.62	16	Sweden
9	Wozniakowski, grzegorz	34	1.80	718	21.12	16	Poland
10	Alonso, covadonga	30	1.59	1,278	42.60	20	Spain
11	Ramirez-medina, elizabeth	30	1.59	477	15.90	12	United States

**Figure 3:**
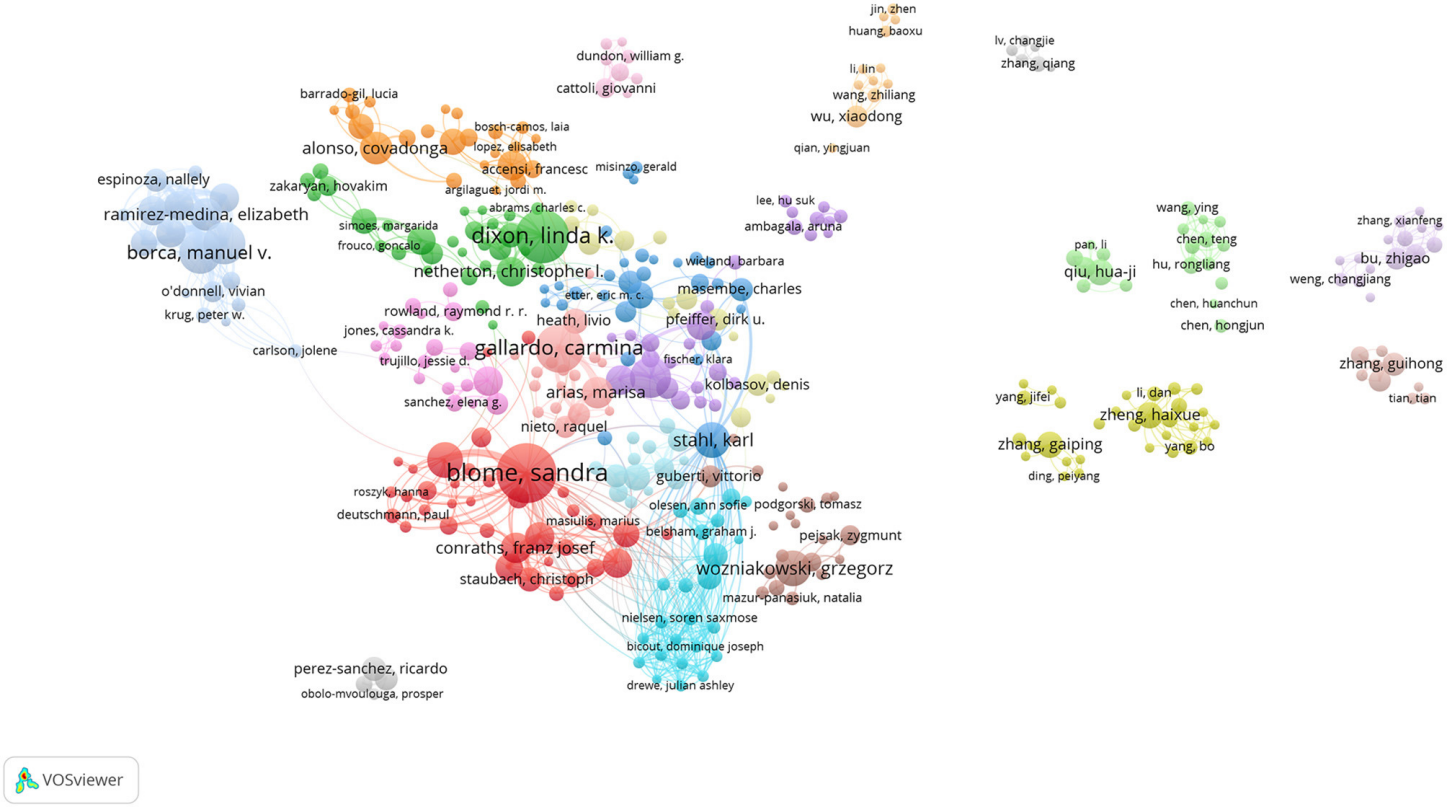
The network map of co-authorship related to ASF.

To highlight the collaboration between the core authors in the field, authors with more than 5 publications are visualized in Figure 3. The size of each node in the figure represents the number of articles published by each author, and the thickness of the connecting line represents the strength of cooperative publishing between authors. Each color represents a cluster, and authors whose nodes are of the same color cooperate closely. We can see that the authors have formed multiple research groups based on their cooperative relationships, indicating that scholars in this field have a certain consensus basis on ASF. Overall, the representative scholars in this field are mainly from Europe and the United States, and they have formed their cooperation networks, and the communication and cooperation between the groups are relatively close. Although Chinese scholars also performed well in this field, only many small cooperative clusters were formed, and no good cooperation network has formed between the various groups.

### 3.3. Most active journals

Statistics on the journals that published the literature show that articles on ASF research have been mainly published in 338 journals in the past two decades. By setting the minimum number of documents to 5, we obtained the co-occurrence map of journals as shown in Supplementary Figure S1. The more published articles, the brighter the color. Conversely, the smaller the number of publications, the darker the color. The top 10 journals published a total of 824 articles, accounting for 43.71%. Table 2 shows the top 10 journals in terms of publication volume, which are mainly professional journals in the field of veterinary sciences and virology, and some interdisciplinary comprehensive journals. Journals that have published more than 100 articles about ASF in the past two decades include *Transboundary and Emerging Diseases* (203, accounting for 10.77%, 21.85 average citation per article, Q1), *Viruses-Basel* (137, accounting for 7.27%, 11.77 average citation per article, Q2), and *Frontiers in Veterinary Science* (106, accounting for 5.62%, 8.66 average citation per article, Q1). In particular, *Transboundary and Emerging Diseases* and *Frontiers in Veterinary Science* are the top journals in the field of veterinary sciences. The former favors content on novel scientific approaches to the causes, control and prevention of cross-border and emerging diseases. The latter is the third most cited journal in the field, covering almost all veterinary sciences such as animal infectious diseases, genomics, veterinary public health, and the exploration of new treatments. The journal with the most average citation is *Virus Research*, with a total of 50 articles published and an average of 60.20 citation per article, indicating that this journal has received more attention in the field of ASF research. Its publications consist primarily of original research papers in the field of viral molecular science, review articles, and special issues on specific topics. Book reviews, editorials and conference presentations are also occasionally accepted.

**Table 2:** The top 10 journals with most publications related to ASF.

**Runk**	**Journals**	**Publications**	**Percentage**	**Citations**	**Average citation**	**H-index**	**IF (2021)**	**Category**	**Quartile in category**	**Region**
1	Transboundary and Emerging Diseases	203	10.77	4,435	21.85	36	4.521	Veterinary sciences	Q1	Germany
2	Viruses-basel	137	7.27	1,613	11.77	20	5.818	Virology	Q2	Switzerland
3	Frontiers in Veterinary Science	106	5.62	918	8.66	18	3.471	Veterinary sciences	Q1	Switzerland
4	Journal of Virology	87	4.62	3,335	38.33	33	6.549	Virology	Q2	United States
5	Pathogens	56	2.97	320	5.71	11	4.531	Microbiology	Q2	Switzerland
6	PLoS One	51	2.71	1,199	23.51	23	3.752	Multidisciplinary sciences	Q2	United States
7	Veterinary Microbiology	51	2.71	1,538	30.16	24	3.246	Veterinary sciences	Q1	Netherlands
8	Virus Research	50	2.65	3,010	60.20	32	6.286	Virology	Q2	Netherlands
9	Preventive Veterinary Medicine	45	2.39	887	19.71	19	3.372	Veterinary sciences	Q1	Netherlands
10	Scientific Reports	38	2.02	940	24.74	20	4.997	Multidisciplinary sciences	Q2	England

Among the top 10 journals with the most publications related to ASF, *Journal of Virology* had the highest impact factor (IF 2021 = 6.549), followed by *Virus Research* (IF 2021 = 6.286) and *Viruses-basel* (IF 2021 = 5.818), and these three journals belong to Q2 of Journal Citation Reports (JCR) virology category. In addition, the three journals with the highest H-index are: *Transboundary and Emerging Diseases* (H-index = 36) followed by *Journal of Virology* (H-index = 33) and *Virus Research* (H-index = 32), their published articles related to ASF had an important influence on this field. The publishing regions of the top 10 most productive journals are all from Europe or the United States, while the Netherlands and Switzerland have 3 each and the United States has 2, indicating that journals from Europe and the United States have been instrumental in advancing this field of research.

### 3.4. Contribution of countries/regions

**Figure 4:**
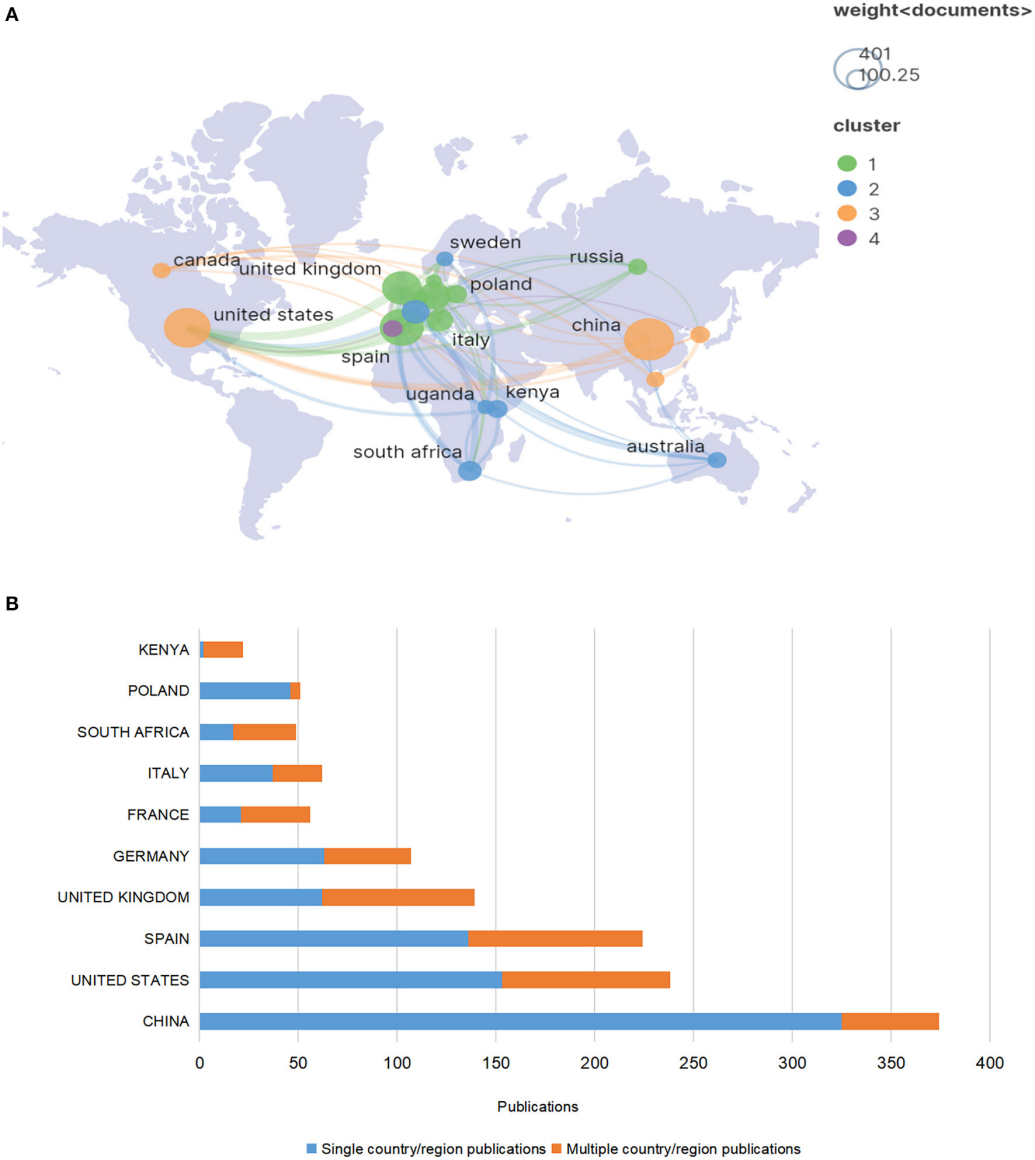
**(A)** The geographical distribution and collaboration map of the top 20 countries/regions with publications related to ASF. **(B)** Multi-country collaboration in the corresponding author's country/region.

To understand the contribution and cooperation of various countries/regions in the field of ASF research, we analyzed the number of publications of 111 countries/regions that have been identified. First, VOSviewer was used to visualize the top 20 countries/regions with the most publications, and then SCImago Graphica version 1.0.23 is used to visualize the geographical distribution, as shown in Figure 4A. The nodes in the figure represent the number of articles published by the countries, and the larger the node is, the more articles are published by the country. The line represents the strength of the association, and the thicker the line is, the greater the number of published articles in cooperation between the two countries. The colors of the nodes represent different clusters, and the cooperation between countries of the same color is relatively close. It can be seen in the figure that countries with high publication volume form four obvious clusters, indicating that there is a stable cooperative relationship between countries. The top 10 countries/regions by publication volume are presented in Table 3. Through the analysis of the data and the visual map, it can be seen that China published the most articles in the field of ASF (401, 4,965 citations, 12.38 average citation per article), but the frequency of cooperation with other countries is not high, and the citation per article are relatively low. The second-ranked United States (353, 7,872 citations, 22.30 average citation per article), third-ranked Spain (312, 11,387 citations, 36.50 average citation per article), and fourth-ranked United Kingdom (252, 9,661 citations, 38.34 average citation per article) have more collaborative publications, indicating that they have frequent academic exchanges in this field.

**Table 3:** The top 10 countries/regions with most publications related to ASF.

**Runk**	**Countries/ regions**	**Publications**	**Citations**	**Average citation**	**H-index**	**Status of the corresponding author's country/region**				
						**Publications**	**Percentage**	**SCP**	**MCP**	**MCP Ratio**
1	China	401	4,965	12.38	36	374	19.84	325	49	13.10
2	United States	353	7,872	22.30	47	238	12.63	153	85	35.71
3	Spain	312	11,387	36.50	58	224	11.88	136	88	39.29
4	United Kingdom	252	9,661	38.34	53	139	7.37	62	77	55.40
5	Germany	167	4,138	24.78	37	107	5.68	63	44	41.12
6	France	125	4,029	32.23	32	56	2.97	21	35	62.50
7	Italy	112	2,546	22.73	30	62	3.29	37	25	40.32
8	South Africa	90	3,386	37.62	31	49	2.60	17	32	65.31
9	Poland	75	1,531	20.41	22	51	2.71	46	5	9.80
10	Kenya	72	1,955	27.15	26	22	1.17	2	20	90.91

SCP, single country/region publications; MCP, multiple country/region publications.

To further show the contribution of each country/region in the field of ASF research, we also counted the distribution of the corresponding author's country/region. China remains the most productive country (374, accounting for 19.84%), followed by the United States (238, accounting for 12.63%) and Spain (224, accounting for 11.88%). In addition, SCP represents the number of publications co-authored by authors from the same country, whereas MCP represents the number of papers co-authored with authors from multiple countries. According to the MCP ratio, Poland, as well as China, have a limited level of international collaboration, whereas Kenya is the most engaged as shown in Figure 4B.

### 3.5. Contribution of institutions

**Figure 5:**
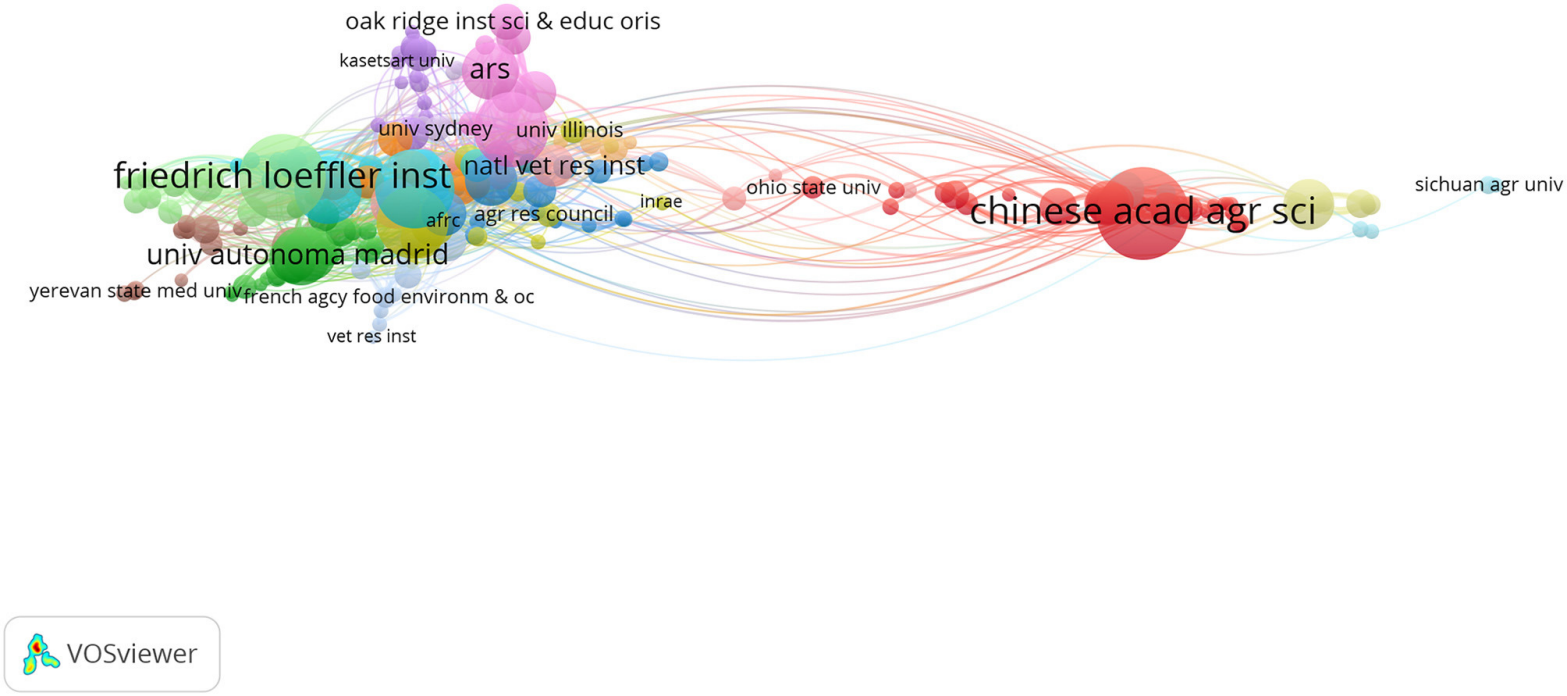
The visualization network map of institutional collaboration.

The publications on ASF research included contributions from 2,021 institutions. According to the number of publications statistics, Table 4 presents the top 10 producing institutions. The top 10 institutions each contributed 718 publications, accounting for 38.09%. Institutions in Europe, Africa, North America, and Asia have made outstanding contributions in this field, indicating that ASF is a global problem that has attracted great attention from scientists all over the world. Although the Chinese Academy of Agricultural Sciences has the most publications (115 papers), the number of citation per article is low (16.30 times). The top H-index value at the Pirbright Institute (H-index = 32) and the highest number of citation per article at the University of Pretoria (43.08 times). The VOSviewer presented the knowledge graph of the institutional collaboration network. By setting the minimum number of documents for one institution as 5, the cooperative network graph of 222 institutions was obtained in Figure 5. There is deep and extensive cooperation between European and American scientific research institutions, while Chinese scientific research institutions cooperate more with domestic institutions. In general, although Chinese scientific research institutions have many publications, the average citation number of articles and the H-index value are not high, and the degree of international cooperation needs to be improved. Among the top 10 institutions, three are from Spain, indicating that the country's scientific institutions have made an outstanding contribution. In addition, scientific institutions in African are an important force in the field of ASF research.

**Table 4:** The top 10 productive institutions ranked by number of publications related to ASF.

**Runk**	**Institutions**	**Publications**	**Percentage**	**Citations**	**Average citation**	**H-index**	**Region**
1	Chinese Acad Agr Sci	115	6.10	1,874	16.30	21	China
2	Friedrich Loeffler Inst	102	5.41	2,700	26.47	27	Germany
3	Pirbright Inst	88	4.67	3,147	35.76	32	England
4	Kansas State Univ	80	4.24	2,081	26.01	27	United States
5	Univ Pretoria	74	3.93	3,188	43.08	31	South Africa
6	Univ Complutense Madrid	66	3.50	2,664	40.36	31	Spain
7	Univ Autonoma Madrid	53	2.81	2,017	38.06	28	Spain
8	ARS (USDA)	50	2.65	1,646	32.92	23	United States
9	CSIC	46	2.44	1,144	24.87	20	Spain
10	Int Livestock Res Inst	44	2.33	1,097	24.93	20	Kenya/Ethiopia

### 3.6. Keyword analysis

#### 3.6.1. High frequency keyword analysis

In a subject area, the topics that scholars are concerned about, having internal connections, and being relatively large in number may be the research hotspots in this field. As a refined expression of research topics and content in academic papers, keywords can reflect the research hotspots in the subject area to a certain extent. The analysis of keywords in the research literature can be used to analyze the evolution of research hotspots.

CiteSpace was used to divide the analysis objects by 1 year per slice, the node type was selected as keyword, and the cosine algorithm was used to select the association strength of network nodes within slices. The threshold (top N) was set as 10, that is, the top 10 of high-frequency keywords in each time slice were extracted. The pathfinding algorithm was chosen in this study, pruning sliced network and pruning the merged network are used to show a clearer co-occurrence network. Table 5 lists the top 20 high-frequency keywords retrieved by CiteSpace to current research, identifying certain established themes and terra incognita in the given research area. Through keyword analysis, in addition to the search terms “African swine fever”, this study also presented keywords related to ASF pathogens, such as “African swine fever virus,” “virus,” “protein,” and “virulence”. Keywords of molecular biological and immunological research related to ASF, such as “gene,” “replication,” “identification,” and “sequence”. Keywords related to ASF epidemiological, such as “epidemiology,” “transmission,” “outbreak,” and “risk factor”. There are also keywords related to the host of ASF, such as “domestic pig” and “wild boar”.

**Table 5:** The top 20 keywords for frequency related to ASF.

**Runk**	**Keywords**	**Frequency**
1	African swine fever	686
2	African swine fever virus	450
3	Virus	293
4	Domestic pig	272
5	Wild boar	204
6	Infection	184
7	Pig	119
8	Gene	100
9	Epidemiology	100
10	Transmission	98
11	Replication	96
12	Protein	85
13	Identification	68
14	Virulence	52
15	Protection	43
16	Outbreak	40
17	Cell	25
18	Risk factor	25
19	Macrophage	21
20	Sequence	20

**Figure 6:**
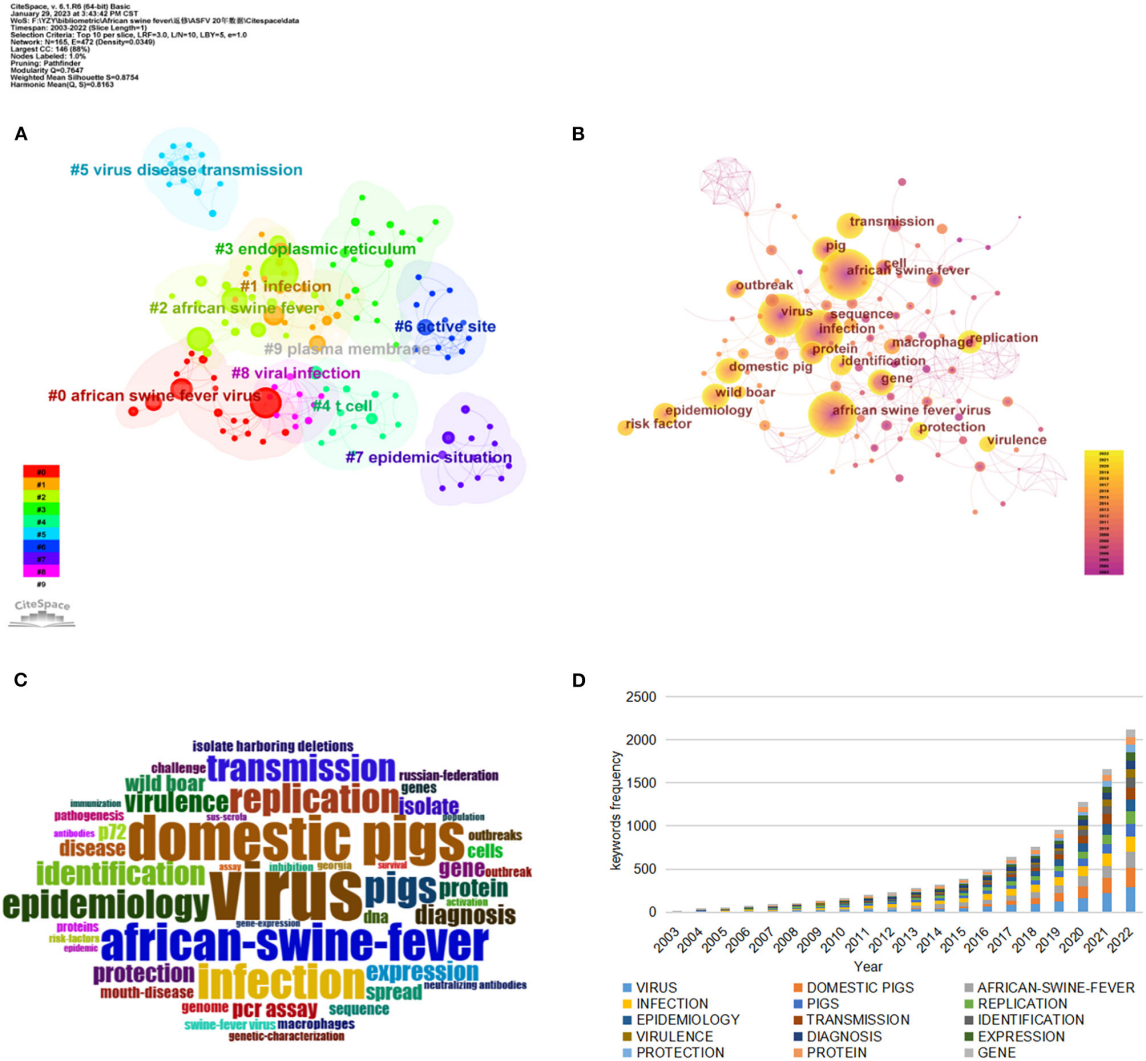
**(A)** The visualization map of keyword clusters related to ASF. **(B)** The visualization map of keyword co-occurrence network related to ASF. **(C)** The keywords cloud. **(D)** Trends of the main keywords over time.

The keyword co-occurrence network in the ASF research field consisted of 165 nodes and 659 links, each node representing a keyword. The larger the node, the higher the frequency of the keyword. Similarly, the color of the node reflects time: the warmer the color, the more recent the time, and the colder the color, the more distant the time. The keyword co-occurrence network diagram was shown in Figure 6B. In addition, to verify the reliability of the core keywords, BiblioShiny was also used to generate the keyword cloud, larger fonts indicate a higher frequency of occurrence (Figure 6C), and it can be seen that the core keywords are roughly the same as those summarized above. The year-by-year evolution of the commonly used keywords is shown in Figure 6D.

#### 3.6.2. Keyword cluster analysis

Keyword clustering is to form some small groups of closely related keywords, to realize the purpose of mining hidden information. It probed certain themes that have been established or may have been overlooked in a particular area of study. In this study, CiteSpace was used to perform keyword clustering analysis based on keyword co-occurrence, using the Log-likelihood Ratio (LLR) algorithm to perform cluster labels extracted from the publications keyword (Figure 6A). In the cluster graph, the presence of cross-clustering indicates that they are closely related. In general, there are two important metrics to evaluate the effect of cluster formation. The modularity Q is used to evaluate the goodness of the network modularity, and a higher value indicates a better cluster obtained by the network. *Q* > 0.3 means that the resulting cluster structure is significant. The cluster silhouette value S is used to measure the homogeneity of cluster members, *S* > 0.5 can be considered as a high consistency of cluster members and the clustering result is reasonable, and *S* > 0.7, the result has high credibility. The *Q*-value of Figure 6A was 0.7647, and the *S*-value was 0.8754, indicating that the clustering structure is significant, and the clustering results are convincing. In the past two decades of ASF studies, 10 meaningful clusters were formed. Sort the clusters from #0 to #9, the smaller the number, the more keywords are included in the cluster, and the details of each cluster are presented in Supplementary Table S1.

#### 3.6.3. Keywords bursts

By using the keyword burst feature of CiteSpace, one can observe the frequent occurrence of certain keywords within a specific period. The information can not only elucidate the dynamics of research hotspots over time but also expose research trends in recent years (27).

**Figure 7:**
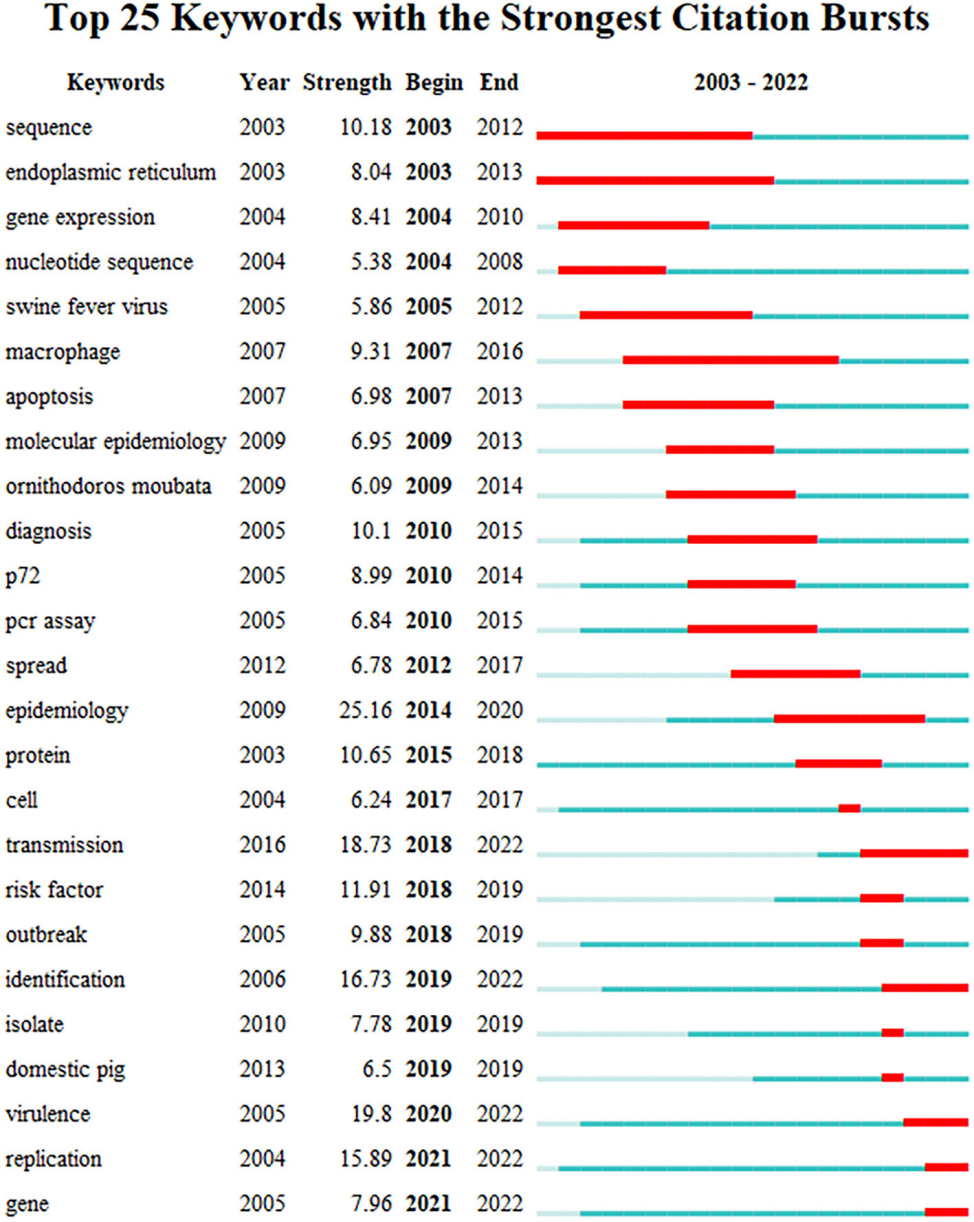
Top 25 keywords with the strongest citation bursts.

Keyword burst detection can be used to explore the sudden increase of research interest in a subject. The information can not only elucidate the dynamics of research hotspots over time but also expose research trends in recent years. The top 25 keywords with the strongest citation bursts were detected as shown in Figure 7. The blue line indicates the year from the beginning to the end of the keyword, and the red line indicates the period when the keyword burst. The stronger the burst strength of the keyword, the more studies related to it. Among the top 25 keywords with the strongest citation bursts, “epidemiology” was the keyword with the greatest burst intensity (Strength = 25.16), a large number of research results are related to this word between 2014 and 2020. At the same time, “endoplasmic reticulum” is the hot keyword with the longest duration (2003–2013). Furthermore, the citation bursts of 5 keywords continued through 2022. Therefore, the 5 burst keywords of “transmission,” “identification,” “virulence,” “replication,” and “gene” reflect the latest research hotspots in the field of ASF.

### 3.7. Co-citation analysis

The behavior of papers citing other papers can be regarded as the flow of knowledge among different research topics and the process of knowledge reorganization to produce new knowledge (44). Citation analysis can be used to evaluate the scientific value and influence of publications in a particular field of research and has a significant impact on the discussion, practice, and future research in this area (45). Moreover, co-citation analysis of cited journals helps to understand key scientific journals in related fields. To understand the most frequently cited papers in the ASF research field and the journals that publish them, as well as the internal relations between them, to obtain the knowledge base source of the research field. In this study, 46,521 references from 12,358 journals were used as data sources to analyze co-cited literature and the journals in which it was published.

#### 3.7.1. Co-citation analysis of cited journals

**Figure 8:**
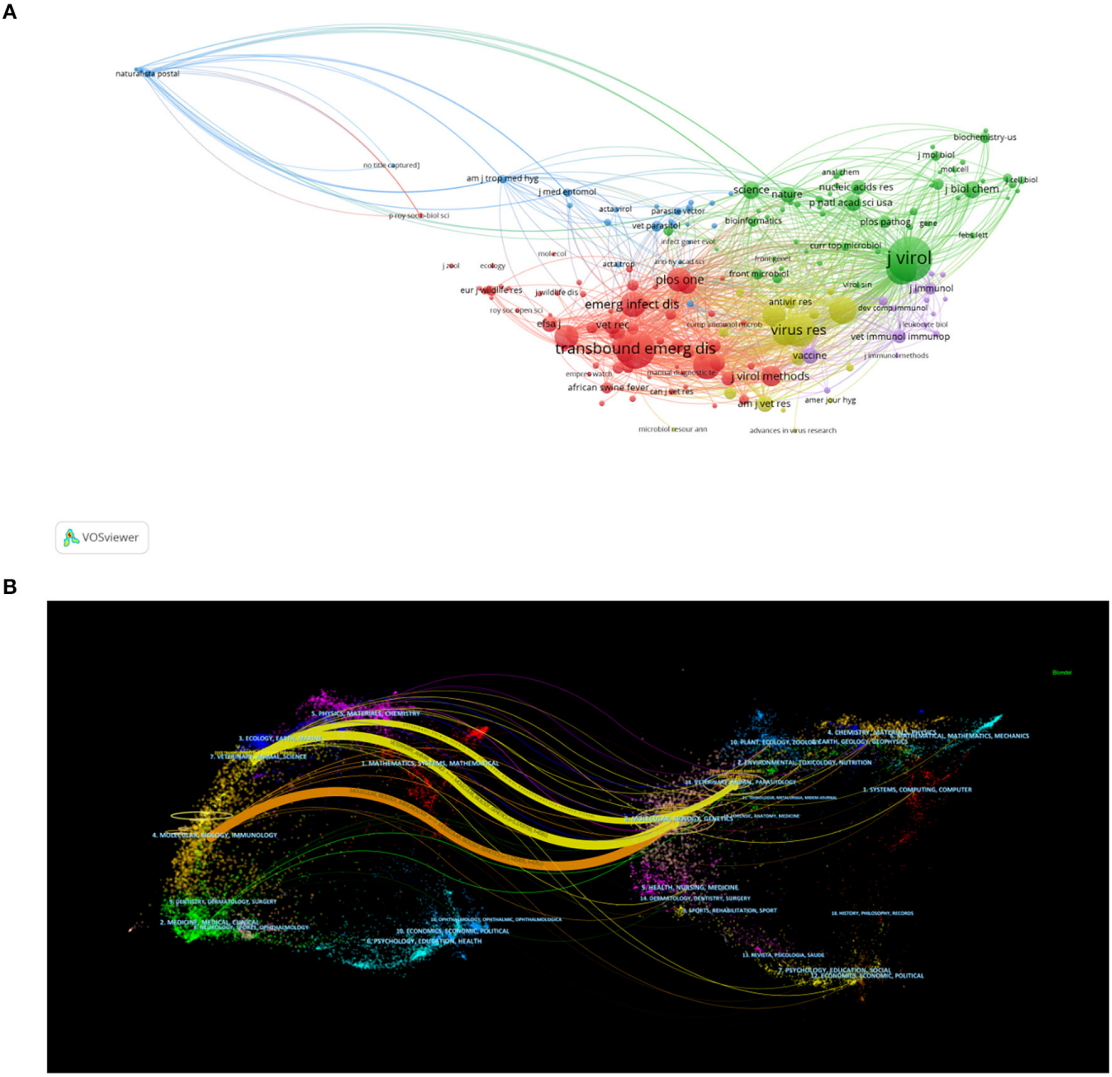
**(A)** The visualization network map of co-cited journals related to ASF. **(B)** The dual-map overlay of journals related to ASF.

VOSviewer was used to analyze the citation network among the cited journals. By setting the minimum number of citations for cited journals to 60, we obtained 183 journals, and the co-occurrence visualization map of co-cited journals was shown in Figure 8A. The node labels on the visualization map describe the co-cited journals, and the lines describe their co-citation relationships. Table 6 lists the top 10 co-cited journals related to ASF. *Journal of Virology* is the most cited journal (5,763 citations, IF 2021 = 6.549, Q2), followed by *Transboundary and Emerging Diseases* (4,125 citations, IF 2021 = 4.521, Q1) and *Virus Research* (3,074 citations, IF 2021 = 6.286, Q2). Additionally, *Emerging Infectious Diseases* is the journal with the highest impact factor (IF 2021 = 16.126), which is the top journal in the field of immunology and has a high authority in the professional field. The dual-map overlay of journals shows the position of a research subject relative to the main research science. Each dot on the map represents a journal, and the labels describe the various research areas covered by all journals. The citing journals are on the left and cited journals are on the right of the map. The colored curve is the citation line, showing the citation relationship, and the width of the curve is closely related to the citation frequency. The longer horizontal axis of the ellipse represents more papers published in the corresponding journal, and the longer vertical axis represents more authors. Figure 8B shows the three main citation paths, indicating that in the field of ASF research, the studies published in veterinary/animal/science categories cited Molecular/Biology/Genetics and veterinary/animal/parasitology Journals. The studies published in Molecular/Biology/Immunology Journals cited Molecular/Biology/Genetics Journals.

**Table 6:** The top 10 co-cited journals related to ASF.

**Runk**	**Journals**	**Citations**	**IF (2021)**	**Category**	**Quartile in category**	**Region**
1	Journal of Virology	5,763	6.549	Virology	Q2	United States
2	Transboundary and Emerging Diseases	4,125	4.521	Veterinary sciences	Q1	Germany
3	Virus Research	3,074	6.286	Virology	Q2	Netherlands
4	virology	2,552	3.513	Virology	Q3	United States
5	Veterinary Microbiology	2,163	3.246	Veterinary sciences	Q1	Netherlands
6	Journal of General Virology	2,090	5.141	Virology	Q2	England
7	Emerging Infectious Diseases	1,793	16.126	Immunology	Q1	United States
8	Archives of Virology	1,700	2.685	Virology	Q3	Austria
9	PLoS One	1,631	3.752	Multidisciplinary sciences	Q2	United States
10	Preventive Veterinary Medicine	1,630	3.372	Veterinary sciences	Q1	Netherlands

#### 3.7.2. Co-citation analysis of cited references

In our study, 46,521 references were used as the analysis object, and the minimum number of citations of cited articles was set to 55, so 145 references were obtained for co-citation analysis. The visual analysis of the 145 most frequently cited articles yield three clusters (Supplementary Figure S2).

Among them, cluster 1 (red) is mainly related to the virulence and pathogenicity of ASFV and its influence on the immune response of the host; cluster 2 (green) is more biased toward the review of ASF and some representative research results, covering the basic knowledge of this research field; and cluster 3 (blue) mostly focuses on genetic characteristics and genotype analysis of ASFV, providing technical support for research. The top five cited references of the three clusters are presented in Supplementary Table S2.

The top three articles in the first cluster were published in *Virus Research, Virology*, and *Journal of General Virology*. Dixon et al. (1) is the most frequently cited (242 times), it summarizes the genome structure of ASFV and basic information about the viral replication mechanism and elaborates on the characteristics and functions of ASFV-encoded genes, providing important theoretical support for other studies. Yáñez et al. presented an analysis of the complete genome of ASFV strain BA71V, and DNA sequence analysis confirmed that ASFV belongs to an independent virus family (46). Chapman et al. (47) determined the genome sequences of two ASFV isolates: a non-pathogenic isolate, OURT88/3, and a highly pathogenic isolate, Benin 97/1. These genome sequences were compared with the BA71 V isolate after tissue culture to study the molecular basis of the differences in pathogenicity of different strains, which provides a basis for further attempts to regulate the differences in viral pathogenesis through genome manipulation.

The second cluster focuses on review articles and some representative reports and studies. The first report of ASF received the most citations in this cluster (256 times), the report titled “On A Form of Swine Fever Occurring in British East Africa (Kenya Colony)” drafted by Montgomery from the records of a veterinary pathology laboratory, then known as the case of East ASF, which was first diagnosed in June 1910. The report described in detail the epidemiological characteristics, clinical diagnosis, and immunotherapy process of the disease at that time, which provided valuable information for follow-up research on ASF (11). A TaqMan-based polymerase chain reaction assay for the detection of ASFV developed by King et al. (48) received the second most frequently citations in this cluster (255 times). As an effective method in the laboratory diagnosis of ASF, it is recommended by the World Organization for Animal Health (49). In particular, the reported occurrence of ASF in China in 2018 also appears in this cluster (50). Moreover, review articles on ASF provide a large amount of comprehensive information for research in this field, present the latest progress and existing problems, and make it easier for readers to understand the dynamics of the discipline.

Publications in the third cluster also received more attention. Rowlands et al. (14) identified the Georgia 2007 isolate of ASFV and determined that it was closely related to genotype II isolates circulating in Mozambique, Madagascar, and Zambia. This paper has been cited 262 times, making it the most cited reference among all subjects analyzed. A review by Costard (51) on how to prevent the global spread of ASF has also been highly cited (260 times). Bastos et al. (52) and Gallardo et al. (53) also received high citations, with 233 and 122 citations, respectively. These articles collectively focus on genetic characterization and genotype analysis of ASFV.

## 4. Discussion

### 4.1. Main outcomes

ASF has been present on the earth for more than 100 years since it was reported for the first time in 1921. In the past 100 years, ASF has caused serious social and economic consequences around the world. To date, there are still many problems in research in this field that urgently need to be solved. Therefore, it has received extensive attention from many scholars around the world. So this is a hot study that is constantly being updated. Based on the information of 1,885 papers on ASF from January 1, 2003 to December 31, 2022, obtained from the WoSCC, this study reviewed the development of this field employing a bibliometric system and visualized the research results through VOSviewer and CiteSpace. We identified the core journals in the field, revealed important authors, contributions of institutions, highly productive countries and their collaborative networks, and explored the keywords, co-cited literature, etc. We also found research hotspots at different times, and keywords involved in these research hotspots are presented.

From the 1,885 published articles related to ASF retrieved from the WoSCC, the number of published articles increased from 16 in 2003 to 400 in 2022. The number of publications on ASF research has increased rapidly over time, indicating that the field is receiving increasing attention. Some renowned scholars have emerged in the research field of ASF, and some author cooperation networks have been established, which play a vital role in promoting the advancement of this field. At present, the author with the most articles on ASF research was Sandra Blome from the Friedrich Loeffler Institute, while Linda Dixon of the Pirbright Institute is the most influential author. The journals that publish ASF-related papers are mainly journals in the professional fields of veterinary science and virology, many of which are top journals in the field of veterinary science. The journal *Transboundary and Emerging Diseases* has published the most publications in this area, it has great academic influence and high professional recognition in the field of industry segmentation. This also hinted at the ASF research has a high professional level in the field of veterinary science. China ranked first in the number of articles published. In 2018, the first ASF epidemic occurred in China and caused a serious impact, which may have caused a research boom among scholars. Another fact is that although China has the most publications, it is not dominant in this field. European countries and the United States have higher H-index and more citation per paper. Similarly, the international cooperation rate of Chinese scholars is much lower than that of scholars from Kenya, South Africa, France, and other countries from the perspective of multi-country cooperation. This shows that Chinese scholars are very important participants in this research field and have great potential for development, but they still need to strengthen cooperation with foreign research institutions and further improve the research level of ASF through complementary advantages. According to the survey of institutions, the Chinese Academy of Agricultural Sciences has contributed the most articles, while the Pirbright Institute has contributed many high-impact articles. And more, the output and quality of scientific institutions in Spain in the field of ASF research are impressive.

### 4.2. Hotspots and frontiers on ASF research

Keywords are indicative words that relate to the content of the research topic, which can reflect the research hotspots to a certain extent. The analysis of the literature related to ASF conducted via CiteSpace revealed important keywords, such as “gene,” “epidemiology,” “transmission,” “replication,” “protein,” “identification,” “virulence,” “protection,” “outbreak,” “cell,” “risk factor,” “macrophage,” “sequence,” etc. With the deepening and development of research, the keywords “transmission,” “identification,” “virulence,” “replication,” and “gene” continued to break out until 2022. It reflects that the research related to these keywords is the latest hotspot in the field of ASF.

It can be inferred from the high frequency keywords such as “epidemiology,” “transmission,” “wild boar,” “domestic pig,” and “outbreak” that a significant area of study for ASF is epidemiological research. Molecular biology is commonly employed by researchers as a pivotal tool for the epidemiological investigation of ASF to analyze the characteristics of viral genes and study the dynamic information of the disease. By collecting ASFV strains from domestic pigs and wild boars, researchers have been able to track the genetic variability of ASFV strains over time and understand the distribution of different genetic variants of the virus (54). Genotyping and phylogenetic analysis of ASFV can be used to determine the likely origin of the virus and to differentiate it from closely related strains (55). Furthermore, when combined with the phylogeographic approach, we can gain a more intuitive understanding of the transmission modes and dynamics of ASF. This information can then be utilized to implement targeted control strategies in specific regions to prevent the spread of this disease (56). The keywords “virus,” “protein,” and “virulence” suggested that the research hotspots were also related to the pathogen of ASF. ASFV is a complex nucleocytoplasmic large DNA virus. The uniqueness and complexity of its structure may be the factors that make it difficult to control (57). Therefore, the study of ASFV structure and protein function is also an important direction in this field. By studying the cryo-EM structure of the ASFV virion, scholars have revealed its important protein structure and the basis of assembly, opening a new way for the development of the ASF vaccine (7, 57, 58). It has also been found that the protein–protein interaction mechanism between ASFV and host pigs is important for studying potential drug targets and predicting the direction of antiviral drugs (59). By understanding the structure and function of certain ASFV proteins, we can gain insight into how to block the development of this virus, as well as provide dissimilar diagnostic strategies to amplify the level of diagnosis (60–62). Additionally, the study on the infection and immune response mechanisms of ASFV is also a crucial research direction, as evidenced by the clustered keywords such as “macrophages,” “infected cells,” “replication,” “immunologics,” and “mechanisms” (as outlined in Supplementary Table S1). The development of ASF may be due to virus-induced changes in the host's immune system, allowing the virus to better adapt to it (63). The mechanisms by which ASFV evades or inhibits the innate immune system involve the regulation of various complex signaling pathways (64). For example, through the inhibition of interferon regulatory factor-3 activation and stimulating interferon gene phosphorylation, the ASFV Armenia/07 virulent strain is capable of impeding the synthesis of IFN-β in porcine alveolar macrophages, thereby evading the host's immune responses (65). Moreover, certain genes of ASFV can regulate the replication of the virus within the host organism. Scholars found that ASFV with deletion of the CD2v and MGF360-505R genes caused significantly less cytopathic effects and apoptosis in PAMs than wild-type ASFV (66). The keywords “recombinant antigen,” “rDNA technology,” “immunization,” and “vaccinology” (shown in Supplementary Table S1) reflect the research focus on vaccines study. Despite the remarkable achievements in ASF research in recent years, we have to face the reality that there is currently not universally accepted safe and reliable ASF vaccine regimen. At present, although researchers have made many explorations into vaccine adjuvants, vaccination methods, and doses, inactivated ASFV vaccines still cannot achieve good protective effects (37, 67). The vaccine research program for ASF is more focused on live attenuated virus vaccines and subunit vaccines. The efficacy of live attenuated vaccines has been established, and research strategies include deleting specific genes, naturally attenuated virus isolates, and cell passages. However, their safety profile is not without risk (68–70). Side effects have been found in vaccinated animals (5). The protective effects of subunit vaccines are also inconsistent due to various reasons, such as vaccination methods and antigen strategies used (71, 72).

It is equally important that the co-citation analysis shows highly co-cited references and highly co-cited journals, which is helpful for subsequent scholars to quickly understand the core knowledge structure and sources of the research field. In conclusion, this study conducted a systematic and comprehensive review of ASF related studies based on the WoS database using a bibliometric approach. Through a quantitative and visual review of ASF research, researchers can accurately grasp the development dynamics and potential trends of ASF research.

## 5. Strengths and limitations

As opposed to traditional literature reviews, bibliometrics is a comprehensive knowledge system that integrates mathematics, statistics, and philology, and pays attention to quantification. Through visualized analysis, it can provide a clear depiction of the development process, research status, and research hotspots of a given field, thereby serving as a reference for further research. However, due to some objective factors, there are certain limitations in this research. First, the samples in this study are only from the Science Citation Index Expanded database in the WoSCC. Although the WoS covers a wide range of journals and is a mainstream source of data in the bibliometrics field, there may still be several articles on this topic that are not counted by WoS. Nevertheless, it is worth noting that WoS is the most frequently employed database in bibliometric studies (73, 74). Second, to ensure the integrity of information in the analyzed literature, the data collected in this study were limited to articles and review articles, and literature types such as meeting reports, books, and case reports were not included. Third, although the software used for bibliometric analysis and the data collected are objective, the subjective nature of the analysis and interpretation cannot be avoided. Finally, recent high-quality studies may not be as widely cited due to their recentness, so influential studies may need to be highlighted by several years of high citation.

## 6. Conclusion

This is the first report that uses bibliometric indicators and information visualization tools to reveal the research status of ASF over the past two decades. It clearly shows the structural changes, research priorities, and knowledge sources of the ASF scientific research field. It will help scholars who are interested in the ASF to further understand the development process of this field and help scholars quickly find the literature, author, or journal they need to cite. Furthermore, predictions based on bibliometric analysis can also provide ideas for future research.

## Data availability statement

The original contributions presented in the study are included in the article/Supplementary material, further inquiries can be directed to the corresponding author.

## Author contributions

LX and ZY designed the research subject and interpreted results. LX analyzed the data and wrote the manuscript. PS, JZha, and WA searched the literature and screened all potentially eligible studies. MY and JZhe reviewed and corrected the manuscript. HL and ZY critically revised the manuscript. All authors contributed to the article and consented to the final manuscript. All authors contributed to the article and approved the submitted version.

## Supplementary material

Supplementary Figure S1 The co-occurrence map of most active journals related to ASF.

Supplementary Figure S2 The co-occurrence network map of co-cited references.

Supplementary Table S1 The keywords co-occurrence cluster list.

Supplementary Table S2 The top 5 cited references in each cluster.
