# Supplementary figures and images for: New perspective on African swine fever: a bibliometrics study and visualization analysis
Source: Front Vet Sci. 2023 May 17;10:1085473. doi: 10.3389/fvets.2023.1085473 (PMC10229902; doi:10.3389/fvets.2023.1085473)

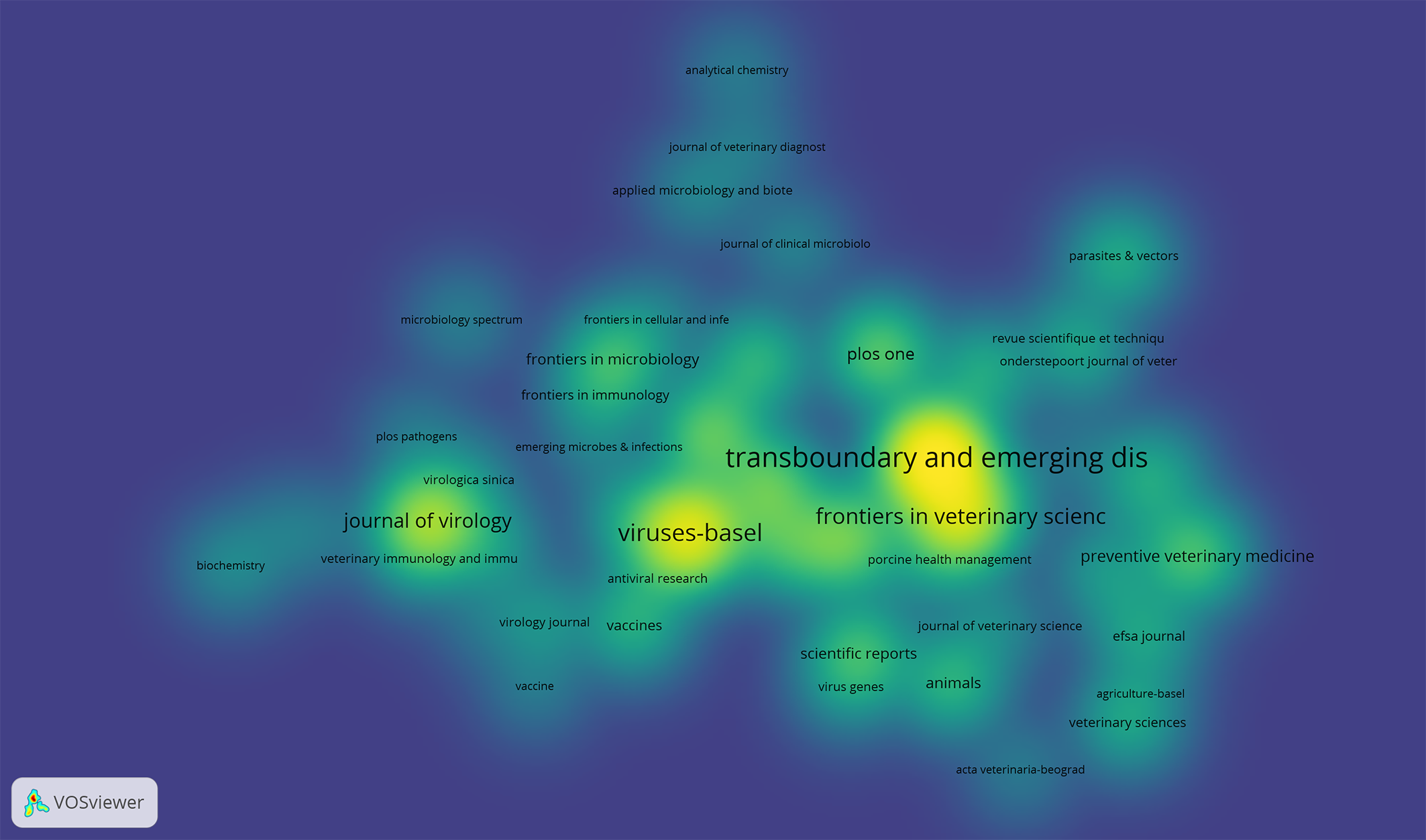

Supplement: Supplementary Figure S1 — The co-occurrence map of most active journals related to ASF. [file Image_1.TIF]

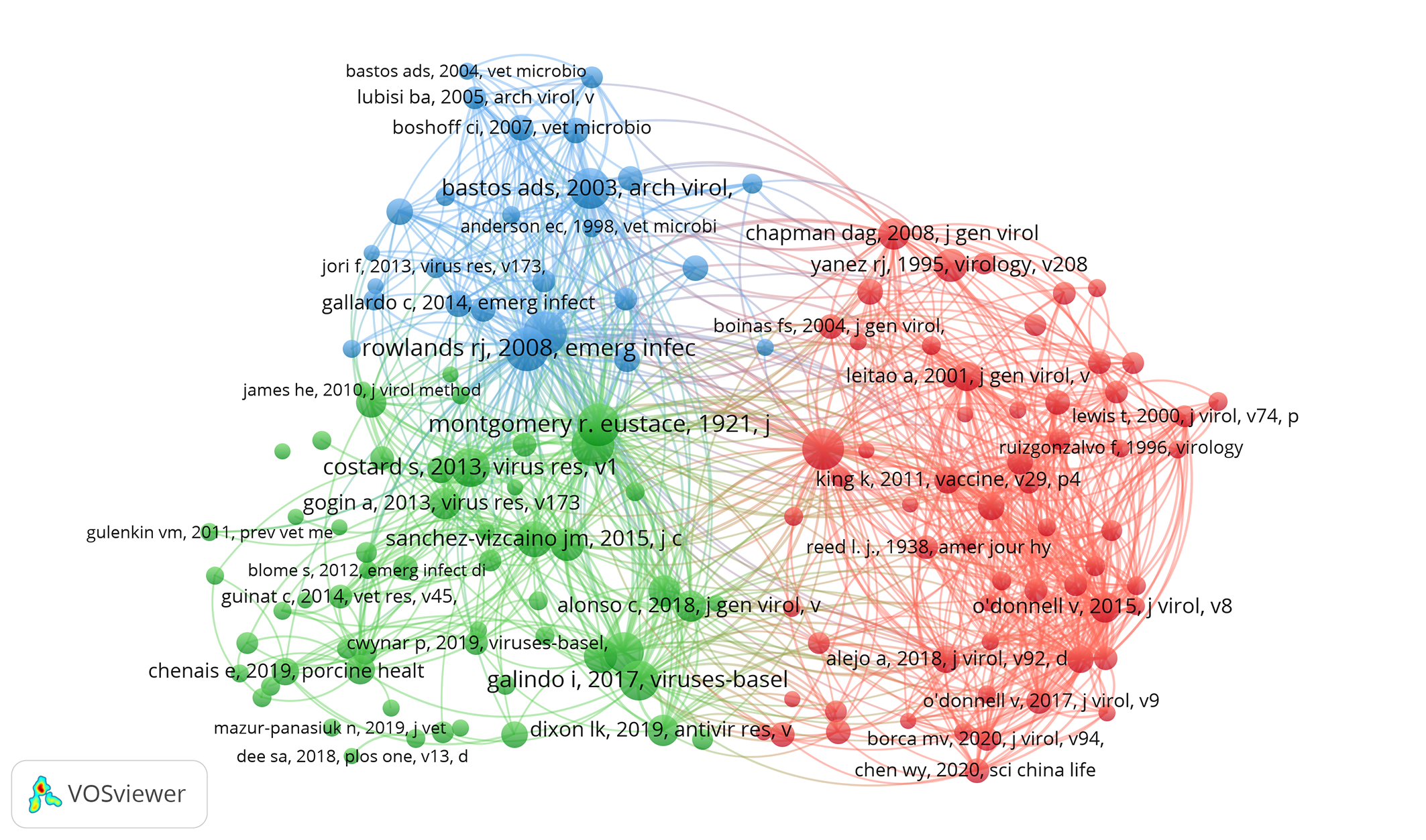

Supplement: Supplementary Figure S2 — The co-occurrence network map of co-cited references. [file Image_2.TIF]
